# Essential oil extracted from Quzhou Aurantii Fructus prevents acute liver failure through inhibiting lipopolysaccharide-mediated inflammatory response

**DOI:** 10.1007/s13659-023-00398-9

**Published:** 2023-10-07

**Authors:** Tian Lan, Wen Wang, De-Lian Huang, Xi-Xi Zeng, Xiao-Xiao Wang, Jian Wang, Yu-Hua Tong, Zhu-Jun Mao, Si-Wei Wang

**Affiliations:** 1 The Joint Innovation Center for Health and Medicine, Quzhou People’s Hospital, The Quzhou Affiliated Hospital of Wenzhou Medical University, No. 100 Minjiang Road, Quzhou, 324000 China; 2 Preventive Treatment Center, Zhejiang Chinese Medical University Affiliated Four-provinces Marginal Hospital of Traditional Chinese Medicine, Quzhou Hospital of Traditional Chinese Medicine, Quzhou, 324000 China; 3 School of Medical Technology and Information Engineering, Zhejiang Chinese Medical University, Hangzhou, 310053 China; 4 Department of Drug Analysis Center, Quzhou Institute for Food and Drug Control, Quzhou, 324000 China; 5 Department of Ophthalmology, Quzhou People’s Hospital, The Quzhou Affiliated Hospital of Wenzhou Medical University, Quzhou, 324000 China; 6 College of Pharmaceutical Sciences, Zhejiang Chinese Medical University, No. 548 Binwen Road, Hangzhou, 310053 China

**Keywords:** Quzhou Aurantii Fructus, Essential oil, Acute liver failure, LPS, Inflammation

## Abstract

Quzhou Aurantii Fructus (QAF) has a long history as a folk medicine and food for the treatment of liver diseases. While our earlier study provided evidence of hepatoprotective properties contained within the flavonoids and limonins constituents in QAF, the potential preventative effects afforded by essential oil components present within QAF remains enigmatic. In this study, we prepared Quzhou Aurantii Fructus essential oil (QAFEO) and confirmed its anti-inflammatory effects on liver inflammation through experimentation on lipopolysaccharide and D-galactosamine (LPS/D-GalN) induced acute liver failure (ALF) mouse models. Using RNA-sequence (RNA-seq) analysis, we found that QAFEO prevented ALF by systematically blunting the pathways involved in response to LPS and toll-like receptor signaling pathways. QAFEO effectively suppressed the phosphorylation of tank-binding kinase 1 (TBK1), TGF-beta activated kinase 1 (TAK1), interferon regulatory factor 3 (IRF3), and the activation of mitogen activated kinase-like protein (MAPK) and nuclear factor-kappa B (NF-κB) pathways in vivo and in vitro. Importantly, QAFEO substantially reduced myeloid differentiation primary response gene 88 (MyD88)- toll-like receptor 4 (TLR4) interaction levels. Moreover, 8 compounds from QAFEO could directly bind to REAL, TAK1, MyD88, TBK1, and IRF3. Taken together, the results of our study support the notion that QAFEO exerts a hepatoprotective effect through inhibiting LPS-mediated inflammatory response.

**Graphical abstract:**

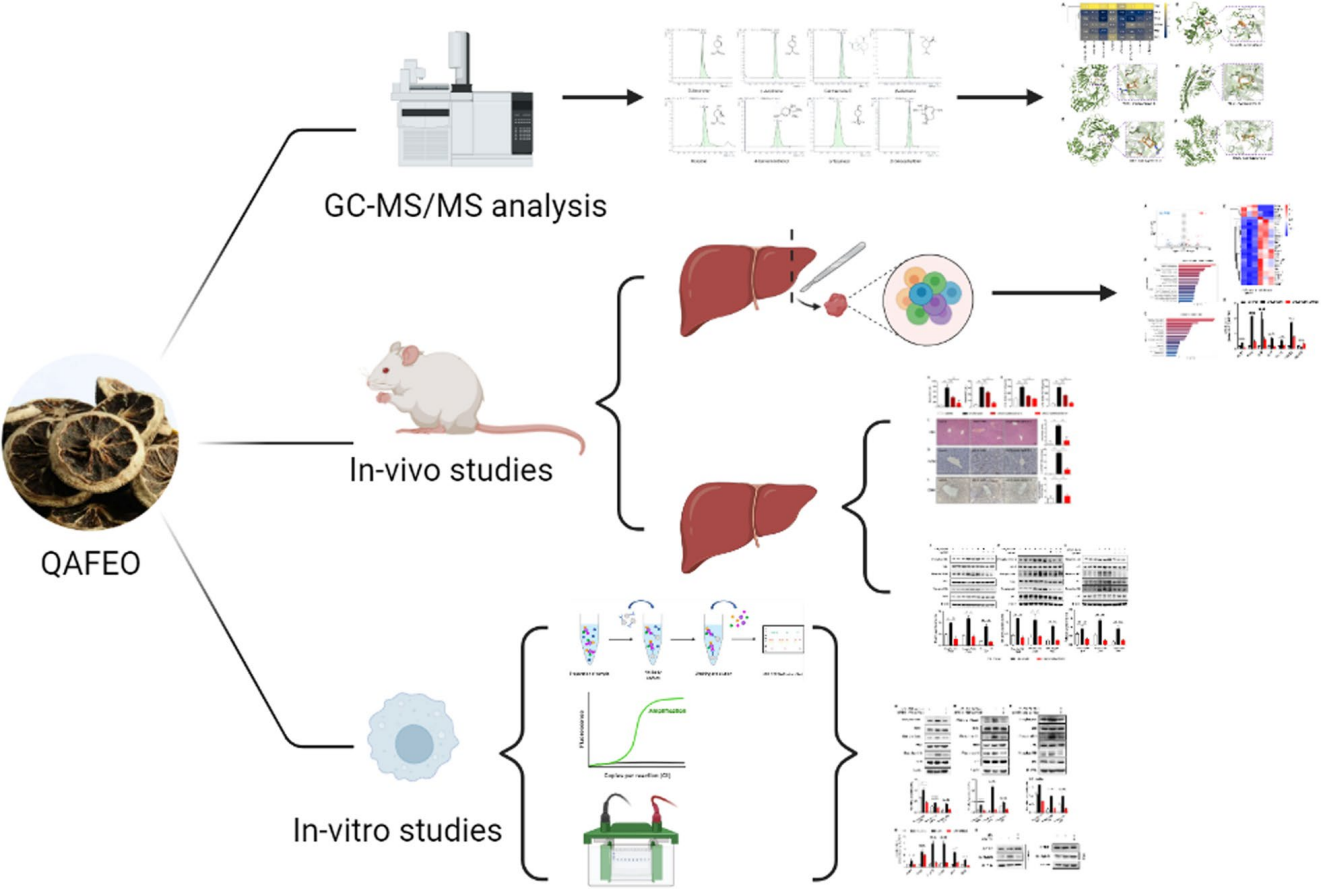

**Supplementary Information:**

The online version contains supplementary material available at 10.1007/s13659-023-00398-9.

## Introduction

Acute liver failure (ALF) refers to the swift decline in hepatic function, which presents as a dramatic clinical syndrome typically resulting from paracetamol toxicity, hepatic ischemia, viral and autoimmune hepatitis, excessive alcohol consumption, and drug overdose [1]. Despite intensive research efforts, ALF remains a dire clinical ailment characterized by high mortality rates and disappointing prognoses. Current clinical management strategies for ALF predominantly involve orthotopic liver transplantation, but this therapeutic option is hampered by inadequate donor organ availability and myriad attendant postoperative complications [2]. Therefore, there is an exigent need to identify novel and efficacious therapeutic approaches for this clinical condition.

Volatile oil, also known as essential oil, is a diverse class of fragrant and volatile oily compounds occurring naturally in plant species. These compounds can be obtained by steam distillation and are typically immiscible with water. Terpenoids and their oxygen-containing derivatives constitute the primary chemical constituents of essential oils [3]. Contemporary pharmacological investigations have revealed that essential oils possess a range of biological properties including anti-inflammatory, anti-oxidative, and antibacterial activities [4, 5]. Citrus fruits possess a distinct anatomical structure, comprised of an oil cell layer, tunica albuginea layer, pulp, and seeds, distinguishing them from other fruit types [6]. Importantly, citrus represents a rich source of essential oils.

*Citrus changshan-huyou* Y.B.Chang, a vernacular plant belonging to the Rutaceae family, arises as a hybridization between *Citrus grandis* Osbeck and *C. sinensis* Osbeck, predominantly prevalent within the confines of Changshan in China [6]. Historically and traditionally, changshan-huyou cultivation in China dates back several hundred years. It has been known that changshan-huyou has a food and medicinal history of more than 300 years. The dried unripe fruit of changshan-huyou is called Quzhou Aurantii Fructus (QAF), which is documented in the “Zhejiang Traditional Chinese Medicine Processing Norms (2015)” [7]. Research findings indicate that QAF primarily constitutes flavonoids, essential oils, limonins, and other constituents [8]. The prior investigations conducted by our team reveal the potential of flavonoids and limonin compounds within QAF to mitigate hepatic injury and suppress inflammation in the liver [9–11]. However, it remains unclear whether the essential oil components present in QAF possess hepatic injury amelioration effects.

In this study, we prepared Quzhou Aurantii Fructus essential oil (QAFEO) and confirmed its anti-inflammatory effects on liver inflammation through experimentation on lipopolysaccharide and D-galactosamine (LPS/D-GalN) induced ALF mouse models. Additionally, our study scrutinized the underlying mechanisms and targets involved in QAFEO’s anti-inflammatory properties.

## Materials and methods

### Reagents and antibodies

Lipopolysaccharide (LPS, #L2630), D-galactosamine (D-GalN, #12,662) and Dimethyl sulfoxide (DMSO, #41,640) were procured from Sigma, St Louis, USA to be used as reagents. Dexamethasone (Dex, #14,648) was purchased from MedChemExpress. The source of 8 standard sample was as follows: Myrcene (CAS: 123-35-3, LOT: X30A11S122905, Shanghai Yuanye Biotech); D-limonene (CAS: 5989-27-5, LOT: 1,012,026,665, Sigma-Aldrich); γ-terpinene (CAS: 99-85-4, LOT: A29J11L119842, Shanghai Yuanye Biotech); 4-Carvomenthenol (CAS: 562-74-3, LOT: J13A9S58322, Shanghai Yuanye Biotech); α-Terpineol (CAS: 98-55-5, LOT: J28J7S9465, Shanghai Yuanye Biotech); β-elemene (CAS: 515-13-9, LOT: J14GB154783, Shanghai Yuanye Biotech); β-Caryophyllene (CAS: 87-44-5, LOT: A05GB156905, Shanghai Yuanye Biotech); Germacrene D (CAS: 23986-74-5, LOT: G367765, Toronto Research Chemicals). Furthermore, a range of antibodies against markers like β-actin (#4967), TBK1 (#3013), phospho-TBK1 (#5483), TAK1 (#5206), phospho-TAK1 (#9339), IRF3 (#4302), phospho-IRF3 (#29,047), IKK (#2370), phospho-IKK (#2697), IκB (#4812), phospho-IκB (#2859), NF-κB p65 (#4764), phospho-NF-κB p65 (#3033), p38 (#9212), phospho-p38 (#9211), JNK (#3708), phospho-JNK (#4668), ERK (#9102), phospho-ERK (#9101), F4/80 (#2370), CD68 (ab125212), TLR4 (ab9105), MyD88 (ab219413), and TRIF (ab13810) were obtained from Cell Signaling Technology or Abcam.

### Preparation of QAFEO

To isolate essential oil from Quzhou Aurantii Fructus, 1 kg of Quzhou Aurantii Fructus was crushed and mixed with 2 L of pure water immediately, followed by extraction using a volatile oil extractor. Extraction conditions entailed heating the mixture to 160 °C for a period of 6 h. Throughout this period, the liquid exhibited slight boiling, generating reflux, and resulting in the continuous flow of a faint yellow volatile oil. This extraction procedure was repeated 10 times, ultimately yielding 80 mL of QAFEO. The obtained QAFEO was carefully sealed, shielded from light, and stored in a cool environment. The technical services were rendered by Academy of Chinese Medical Science, Zhejiang Chinese Medical University (Hangzhou, China).

### GC-MS/MS analysis of QAFEO

**Fig. 1:**
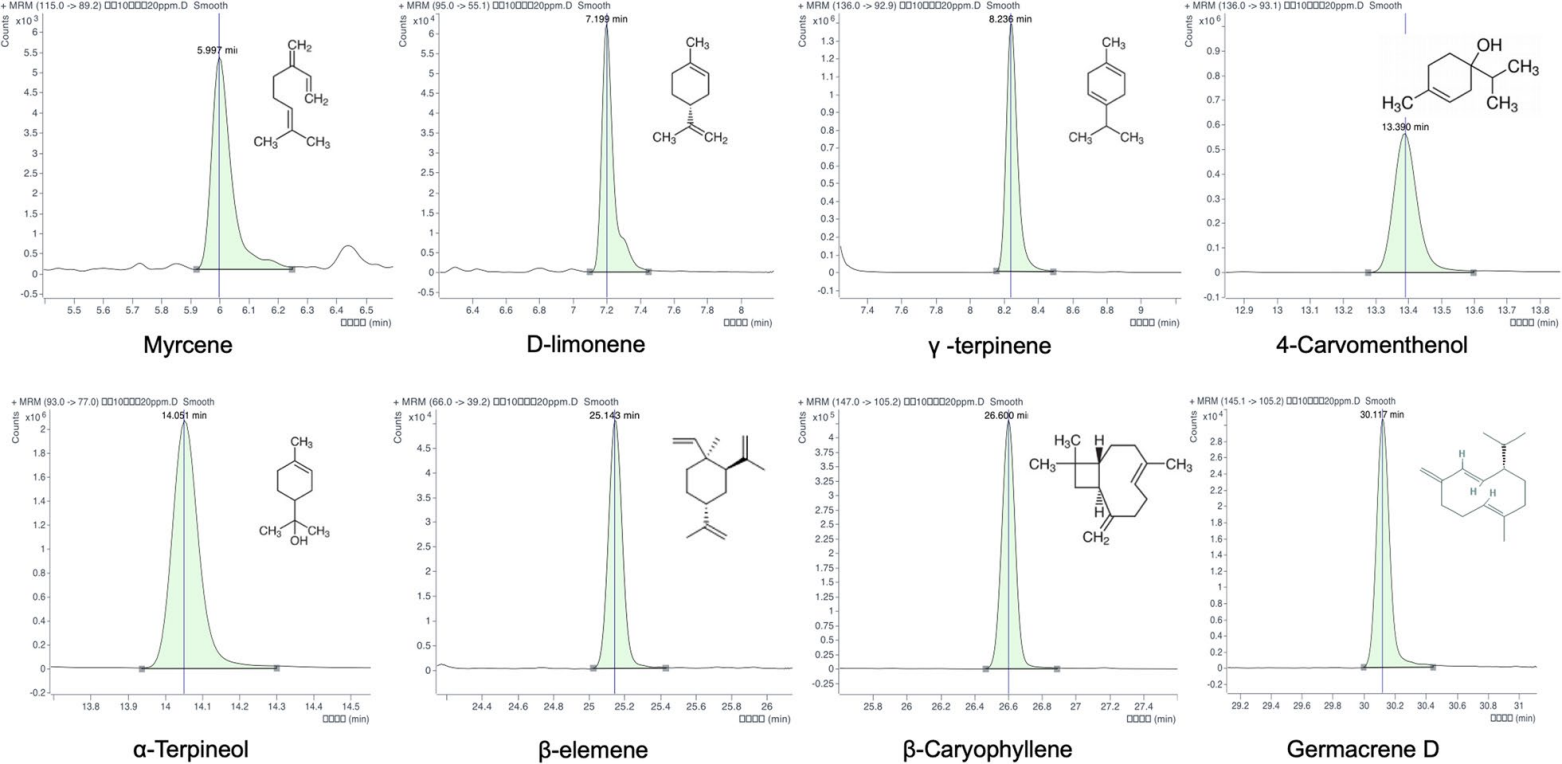
The chemical compositions of QAFEO were analyzed and identified with GC-MS/MS.

The analysis and separation of QAFEO were accomplished using Agilent 7000D gas chromatography-tandem mass spectrometry (GC-MS/MS). Specifically, the QAFEO sample was separated on a 19,091 S-431 HP-5 MS column with dimensions of 15 m × 250 μm × 0.25 μm. Furthermore, the ion source temperature was set to 280 °C, while the inlet temperature was maintained at 260 °C. We employed prescribed operating conditions consisting of a gradual increase from 70 to 150 °C at a rate of 2˚C/min, followed by escalation from 150 to 240 °C at a rate of 6 °C/min and held for 1 min, finally increasing to 300 °C at a rate of 25 °C/min. The samples were injected at a shunt ratio of 1: 3, with 1 µL of the samples utilized for injection. Figure 1; Table 1 display the concentration of each constituent and its corresponding chromatogram. 8 standard samples were injected into the system and an external standard method was used to qualify the test samples.

**Table 1:** GC-MS/MS analysis of QAFEO

No	Retention time (min)	Ingredient name	Concentration (mg/g)
1	5.997	myrcene	21.85
2	7.199	D-limonene	93.18
3	8.236	γ -terpinene	15.74
4	13.390	4-carvomenthenol	7.00
5	14.051	α-terpineol	13.58
6	25.143	β-elemene	28.31
7	26.600	β-caryophyllene	6.81
8	30.117	germacrene D	12.48

### Animal experiment

The proposed experimental protocol was authorized by the Animal Ethics Committees of Quzhou People’s Hospital on December 15th, 2022 and assigned Ethical Approval No. QZRMYY-20221216-001. Gempharmatech Co., Ltd, Jiangsu, China provided male BALB/c mice (22–25 g) that were maintained under standard conditions with access to both distilled water and pelleted food ad libitum. Three groups of eight mice each were randomly assigned in the drug toxicity experiment: control group received oral saline administration for five days, QAFEO-50 group received oral administration of 50 mg/kg/day QAFEO for five days, and QAFEO-100 group received oral administration of 100 mg/kg/day QAFEO for five days. For ALF experiment, four groups of eight mice each were randomly assigned in the experiment. The ALF mouse model was established by administering an intraperitoneal injection (i.p.) of LPS (6 µg/kg) and D-GalN (800 mg/kg). To assess the effects of QAFEO treatment, low dose (50 mg/kg body weight) and high dose (100 mg/kg body weight) of QAFEO were administered once daily for 5 consecutive days prior to administration of LPS/D-GalN. The mice were sacrificed 8 h after being injected with LPS/D-GalN.

### Serum and liver biochemistry testing

Serum and liver alanine aminotransferase (ALT), as well as aspartate aminotransferase (AST) levels, were quantified via a biochemical analyzer in conformity with the manufacturer’s instructions obtained from Nanjing Jiancheng Bioengineering Institute in Nanjing, China.

### Cytokine activity by ELISA

The quantification of inflammation-associated cytokines, including IL-6 (CAT: PI326), TNF-α (CAT: PT512), and MCP-1 (CAT: PC125), in the serum of ALF mice and the supernatant of the LPS-induced cell model was conducted using ELISA kits obtained from Beyotime, China. The experimental procedures were carried out in accordance with the provided guidelines. The absorbance at 450 nm was measured using a Synergy Microplate Reader (BioTek Instruments, Inc.).

### Histopathological analysis

The liver tissues under investigation were subjected to fixation within 10% formalin, followed by embedding into paraffin and subsequent cutting into sections measuring 5 μm in thickness. Hematoxylin and eosin (H&E) staining was then utilized for the samples in question. Evaluation of the liver injury score was performed via blinded assessment of the observed staining intensity, with scoring being conducted upon a scale that ranged between 0 and 10.

### Immunohistochemical staining

The sections underwent deparaffinization using xylene, followed by rehydration via a series of graded alcohol. Antigen retrieval was achieved through heating for a duration of fifteen minutes. Treatment with 3% H_2_O_2_ then proceeded for the tissue sections, alongside incubation with blocking goat serum for one hour. Overnight incubation of the sections took place with anti-F4/80 and anti-CD68 at a temperature of 4 °C. Thereafter, the MaxVision HRP-Polymer anti-Rabbit IHC Kit, sourced from MXB Biotechnologies in China, was employed. The proportion of macrophages that had been immunohistochemically stained was expressed as an interval ranging between 0% and 100%.

### RNA sequencing and data collection

Total RNA was extracted from liver tissues in LPS/D-GalN group and LPS/D-Gal + QAFEO group by using Trizol (#DP424, Tiangen, China). Moreover, RNA quantity and purity were assessed utilizing a NanoDrop ND-1000 instrument (NanoDrop, Wilmington, DE, USA). Furthermore, RNA integrity was determined by employing an RNA Nano 6000 assay kit and a Bioanalyzer 2100 system (Agilent, CA, USA). Subsequently, the prepared libraries (with a size of 300 ± 50 bp) were sequenced in a paired-end configuration (2 × 150 bp) via an illumina Novaseq™ 6000 (Illumina Inc., San Diego, CA, USA). Differential gene expression analysis was performed using the criteria of fold change > 2 or fold change < 0.5 and *p* < 0.05 to define differentially expressed genes (DEGs). All technical services were rendered by the LC Biotech Corporation (Hangzhou, China).

### Network analysis of potential targets for ALF

Targets associated with ALF were primarily sourced from prominent databases including Online Mendelian Inheritance in Man (OMMI; https://www.omim.org), GeneCards (https://www.genecards.org), Therapeutic Target Database (TTD; http://db.idrblab.net/ttd/) and DisGeNET (https://www.disgenet.org/) databases. From the GeneCards database, targets surpassing a relevant score threshold of > 4.111 732 721 were meticulously selected. To ensure data integrity, the target information obtained from the four databases was amalgamated, eliminating any duplicate entries. To identify potential targets for ALF treatment, the differentially expressed genes from the RNA-seq analysis were overlapped with the ALF disease targets via Venny 2.1.0 (https://bioinfogtp.cnb.csic.es/tools/venny/index.html), subsequently visualized using a Venn diagram to depict the intersection.

### GO and KEGG enrichment analysis

To conduct functional enrichment analysis, we harnessed the hub genes derived from network analysis. Leveraging the prowess of the clusterProfiler (version 4.2.2), enrichplot (version 4.2.2), and ggplot2 (version 4.2.2) packages in R software, we scrutinized and graphically depicted the biological processes (BP) encompassing Gene Ontology (GO) terms, as well as delved into the intricate pathways housed within the Kyoto Encyclopedia of Genes and Genomes (KEGG). All profoundly relevant pathways or terms were meticulously sieved with a stringent adjusted *p*-value threshold of < 0.01.

### Molecular docking

In this study, eight compounds were downloaded from the PubChem database as ligands with their corresponding SDF-format files. These SDF-files were further converted to *mol2 format utilizing Openbable software. Furthermore, the 3D structure of the core target in *PDB format was procured from RCSB PDB data as a receptor of interest. Additionally, prior to molecular docking, dehydration and hydrogenation of the protein were performed using pyMOL software. Auto Dock software was employed for converting the compound and target protein format to *pdbqt format followed by molecular docking using Vina. Finally, the results were visualized with PyMOL software.

### Cell culture

The RAW264.7 cell line was cultivated within RPMI-1640 medium, which had been supplemented with 10% FBS (BBI Life Sciences Corporation, Shanghai, China), as well as 100 U/mL of streptomycin and 100 U/mL of penicillin. Cultivation was undertaken at a temperature of 37 °C within an atmosphere containing 5% CO_2_. Upon reaching confluence, the cells were harvested via employment of Gibco’s trypsin solution consisting of 0.25% Trypsin-EDTA.

### Co-immunoprecipitation

For co-immunoprecipitation, extracts were treated with antibodies against TLR4 for a duration of 4 h, followed by precipitation using protein G-Sepharose beads overnight at 4 °C. The levels of MyD88 and TRIF were subsequently detected through immunoblotting using antibodies.

### Real-time qPCR

Total RNA was obtained through an extraction process using the Trizol reagent (Tiangen Biotech Co. Ltd., China), after which cDNA was reverse transcribed from total RNA by means of cDNA Synthesis Kits (Thermo Scientific, USA). Subsequent to the undertaking of these procedures, quantitative real-time PCR was carried out with the utilization of SGExcel FastSYBR Master (Sangon Biotech, Shanghai, China) via the LightCyclerR 480 Quantitative PCR System (Roche, USA). The primers utilized were synthesized by Sangon Biotech and may be referenced within Table 2. The relative gene quantities were determined employing the 2^−ΔΔCt^ method, the normalization of which was conducted utilizing the expression levels of GAPDH.

**Table 2:** The primers used in our current study

Gene	5′-3′ forward strand	5′-3′ reverse strand
*Cd14*	GAAGCAGATCTGGGGCAGTT	CGCAGGGCTCCGAATAGAAT
*Tnf-α*	AGCCGATGGGTTGTACCTTG	ATAGCAAATCGGCTGACGGT
*Ccl3*	TTCTCTGTACCATGACACTCTGC	CGTGGAATCTTCCGGCTGTAG
*Ccl4*	TTCCTGCTGTTTCTCTTACACCT	CTGTCTGCCTCTTTTGGTCAG
*Cxcl9*	CGGACTTCACTCCAACACAG	TAGGGTTCCTCGAACTCCAC
*Cxcl10*	GGATCCCTCTCGCAAGGA	ATCGTGGCAATGATCTCAACA
*Nlrp1b*	GGCTCTCTGATGCCCAACTTA	ACTGATAGAGGAGACCCCACTC
*Gapdh*	TGAGGCCGGTGCTGAGTATGT	CAGTCTTCTGGGTGGCAGTGAT

### Western blot analysis

Tissues or cells were disrupted through a process of lysis using 1×SDS buffer containing phenylmethylsulfonyl fluoride (PMSF), phosphatase inhibitor and protease inhibitor. The separated protein was subjected to sodium dodecyl sulfate polyacrylamide gel electrophoresis (SDS-PAGE) and then transferred onto polyvinylidene fluoride (PVDF) membranes, with the determination of concentration carried out by means of the Biotek Synergy H1 microplate reader. Having been blocked with 0.1% casein for 1 h at room temperature, the protein underwent incubation with primary antibodies overnight at 4 °C, as well as subsequent incubation with secondary antibodies for 1 h at room temperature. Finally, detection was performed by employing Tanon 4200SF system (Tanon Biotechnology, Shanghai, China). Quantification of band intensity was achieved using ImageJ software.

### Data analysis

The experimental results were expressed as mean ± standard deviation (SD). The statistical analysis was conducted with GraphPad Prism 9 software (GraphPad Software, La Jolla, CA, USA). Furthermore, for comparisons involving more than two groups, the Student-Newman-Keuls test was applied, while the Student’s t-test was employed for two-group comparisons; both tests were executed using one-way ANOVA. Statistically significant differences were noted at *p* < 0.05.

## Results

### GC-MS/MS analysis of QAFEO

The identification and quantification of various chemical compounds within the QAFEO were investigated through gas chromatography-tandem mass spectrometry (GC-MS/MS) analysis. The result of GC-MS/MS analysis revealed the presence of eight primary components in QAFEO, namely myrcene, D-limonene, γ-terpinene, 4-carvomenthenol, α-terpineol, β-elemene, β-caryophyllene, germacrene D (Fig. 1). Detailed information concerning the specific retention times and contents of these eight compounds can be found in Table 1.

### QAFEO protects against LPS/D-GalN induced ALF and inhibits inflammatory cell infiltration in mice

**Fig. 2:**
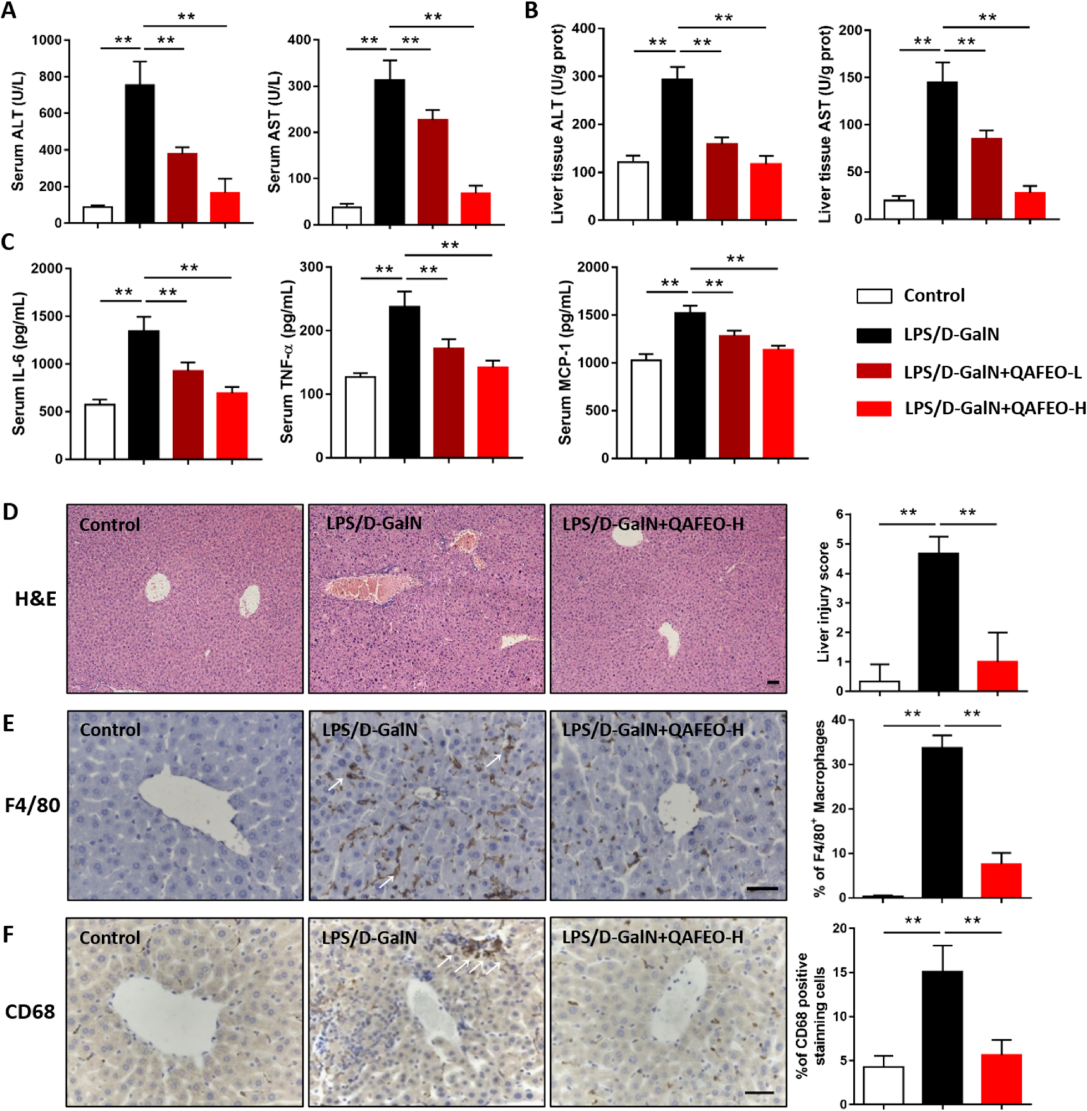
The effect of QAFEO on liver function and inflammatory cell infiltration in LPS/D-GalN-induced mice. Mice were challenged by LPS/D-GalN with or without different dosages of QAFEO treatment. **A**, **B** The levels of ALT and AST from the serum and the livers. Data were expressed as the mean ± SD (n = 8). **C** Serum levels of IL-6, TNF-α and MCP-1 after QAFEO administration in ALF mice model. Data were expressed as the mean ± SD (n = 6). **D** H&E staining of liver sections. Liver injury score was on the right. **E**, **F** Representative images of immunohistochemistry staining for F4/80 and CD68. The calculation of F4/80 and CD68 positive cells was on the right. Scale bar = 300 μm. Data were expressed as the mean ± SD (n = 5). ^**^p < 0.01

Initially, in vivo toxicity assays were performed to evaluate the safety profile of QAFEO. Mice were subjected to treatment with two different doses of QAFEO (50 or 100 mg/kg) for five consecutive days. The outcomes demonstrated that QAFEO didn’t cause any pathological alterations in the liver, as well as no elevation in the liver function markers (Additional file 1: Fig. S1A, B), indicating that both concentrations are safe for in vivo usage. Subsequently, we aimed to examine the potential hepatoprotective effects of QAFEO by establishing a mouse model of acute liver failure (ALF) through LPS and D-GalN induction. Our data revealed that QAFEO significantly reduced serum ALT and AST levels, as well as liver tissue of ALF mice with varying dosages of QAFEO (Fig. 2A, B). Since proinflammatory mediators play vital roles in LPS/D-GalN-induced hepatic damage, we measured serum levels of IL-6, TNF-α and MCP-1. Results showed that low or high dose of QAFEO could significantly decrease serum IL-6, TNF-α and MCP-1 levels after LPS/D-GalN treatment (Fig. 2C). Moreover, high-dose QAFEO effectively alleviated liver histopathological lesions (Fig. 2D) while also considerably reducing liver inflammatory cell infiltration, as demonstrated through F4/80 and CD68 immunohistochemical staining in ALF mice (Fig. 2E, F). Collectively, our analysis suggests that QAFEO exerts a potent hepatoprotective effect against acute liver injury and inhibits hepatic inflammation.

### QAFEO prevents ALF by systematically blunting the pathways involved in inflammation-related signaling pathways

**Fig. 3:**
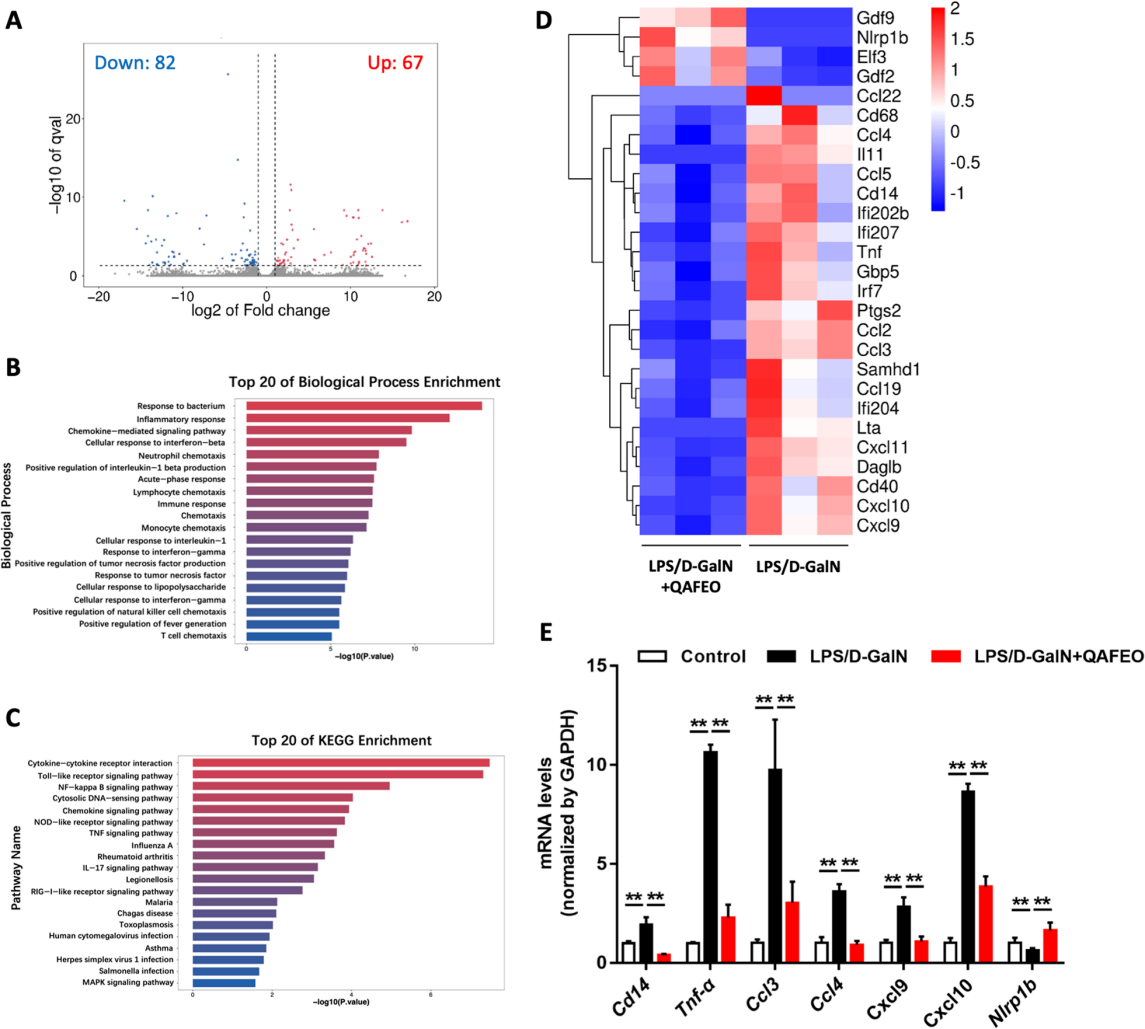
RNA-seq analysis revealed the key differential targets in QAFEO-treated mice of ALF. **A** Volcano plot of DEGs. Compared with the LPS/D-GalN group, 67 genes were up-regulated, and 82 genes were down-regulated in the LPS/D-GalN + QAFEO-H group. FC > 2, *p* < 0.05. **B** Top 20 of GO (biological process) enrichment. **C** Top 20 of KEGG enrichment. **D** Heatmaps of gene expression profiles related to inflammation pathways. n = 3 per group. **E** RT-qPCR analysis of genes involved in the inflammation pathways. Data were expressed as the mean ± SD (n = 3). ^**^p < 0.01

To systematically investigate the potential protective effects of QAFEO on ALF, we conducted RNA-sequencing (RNA-seq) analysis on livers from ALF mice and QAFEO-treated ALF mice. Our findings revealed that compared to LPS/D-GalN-treated mice, administration of QAFEO to ALF mice resulted in differential expression of 67 up-regulated and 82 down-regulated genes (Fig. 3A). Subsequently, further gene ontology (GO) and Kyoto Encyclopedia of Genes and Genomes (KEGG) pathway analysis corroborated the inflammation-related toll-like receptor and NF-κB signaling pathways as being involved in the QAFEO-mediated effects (Fig. 3B, C). In addition, the RNA-seq data was validated by real-time quantitative polymerase chain reaction (RT-qPCR), which indicated significant modulation of gene expression implicated in these inflammatory pathways (Fig. 3D, E). Taken together, our findings suggest that QAFEO may confer protection against ALF through its modulatory effect on inflammation-related signaling pathways.

### QAFEO suppresses LPS-mediated inflammatory response in vivo

**Fig. 4:**
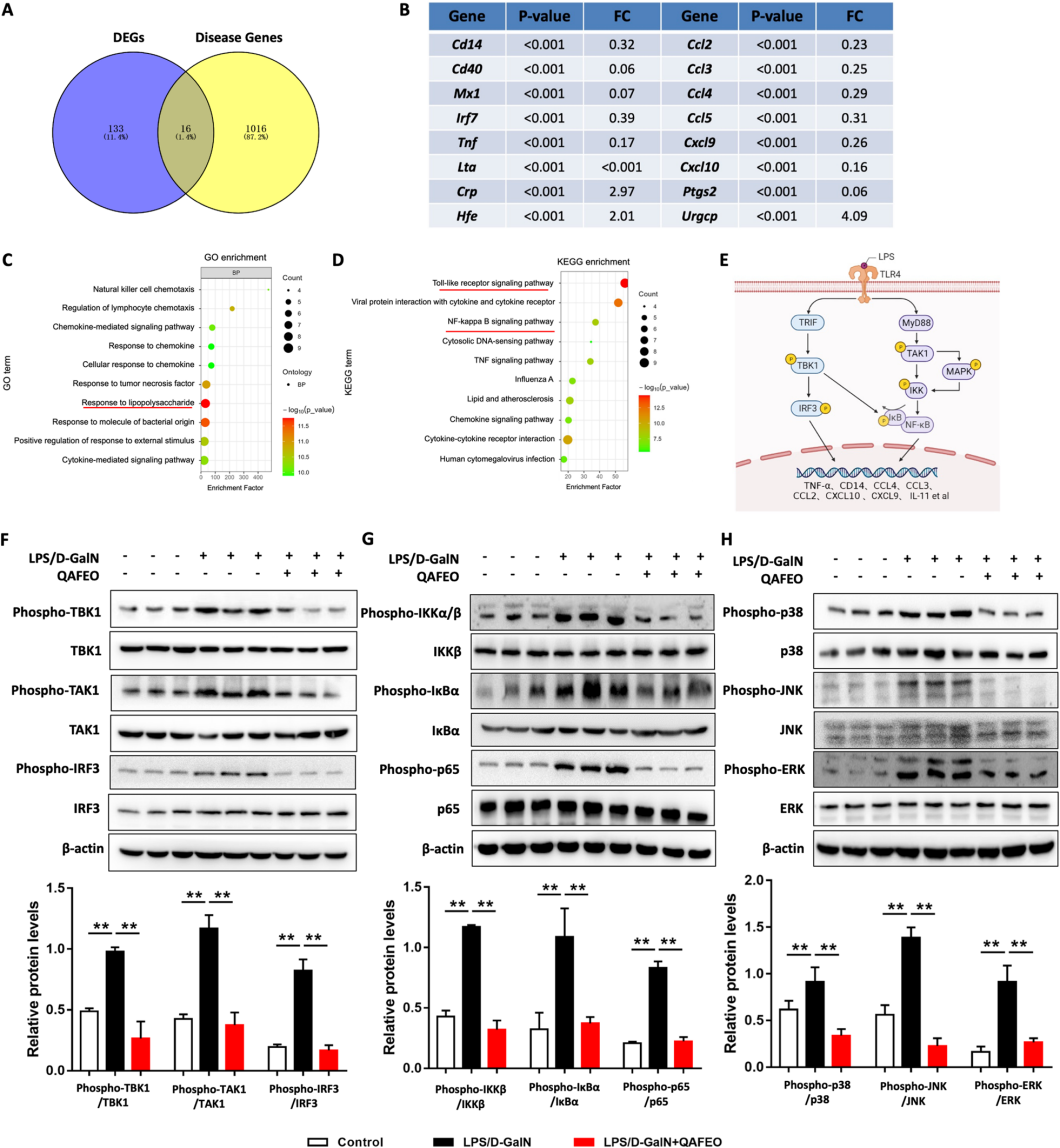
Effects of QAFEO on the expression of inflammatory cytokines and LPS-mediated inflammatory signaling pathways in mice. **A** Venn diagram of differentially expressed genes (DEGs) and disease targets for ALF. **B** The overlap of DEGs in (A). **C** GO enrichment analysis. **D** KEGG enrichment analysis. **E** The accompanying schematic delineates the LPS-induced innate signaling mechanism of Toll-like receptor-4 (TLR4), culminating in the activation of inflammatory factors. **F**–**H** The protein expressions of phospho-TBK1, TBK1, phospho-TAK1, TAK1, phospho-IRF3, IRF3, phospho-IKKα/β, IKKβ, phospho-IκBα, IκBα, phospho-p65, p65, phospho-p38, p38, phospho-JNK, JNK, phospho-ERK and ERK were detected by western blotting. Data were expressed as the mean ± SD (n = 3). ^**^p < 0.01

To elucidate the therapeutic targets of QAFEO in ALF, we utilized differentially expressed genes (DEGs) to connect with disease targets of ALF (Fig. 4A). Notably, several inflammatory factors such as *Tnf*, *Ccl2*, *Ccl3*, and *Cxcl9*, were identified as potential targets of QAFEO regulation (Fig. 4B). GO and KEGG enrichment analyses revealed a significant involvement of QAFEO in the lipopolysaccharide (LPS)-induced inflammatory response and toll-like receptor signaling pathway (Fig. 4C, D). Toll-like receptor 4 (TLR4), which forms the core of the mammalian innate immune system and plays a vital role in LPS-mediated inflammation, mediates inflammatory signals through either MyD88-dependent or TRIF-dependent pathways (MyD88-independent pathways) in innate immunity (Fig. 4E). Intriguingly, our results demonstrated that QAFEO effectively suppressed the phosphorylation of TBK1, TAK1, and IRF3 in liver of ALF mice (Fig. 4F). Moreover, in vivo experiments revealed that QAFEO treatment notably inhibited the expression of phosphorylated proteins of IKK, IκBα, p65, p38, JNK, and ERK, thereby implicating the downregulation of MAPK and NF-κB pathways by QAFEO (Fig. 4G, H). Taken together, these findings suggest that QAFEO regulates the LPS-mediated inflammatory signaling pathway in mouse models of ALF.

### QAFEO inhibits LPS-mediated inflammatory signaling pathways and reduces the level of MyD88-TLR4 interaction in vitro

**Fig. 5:**
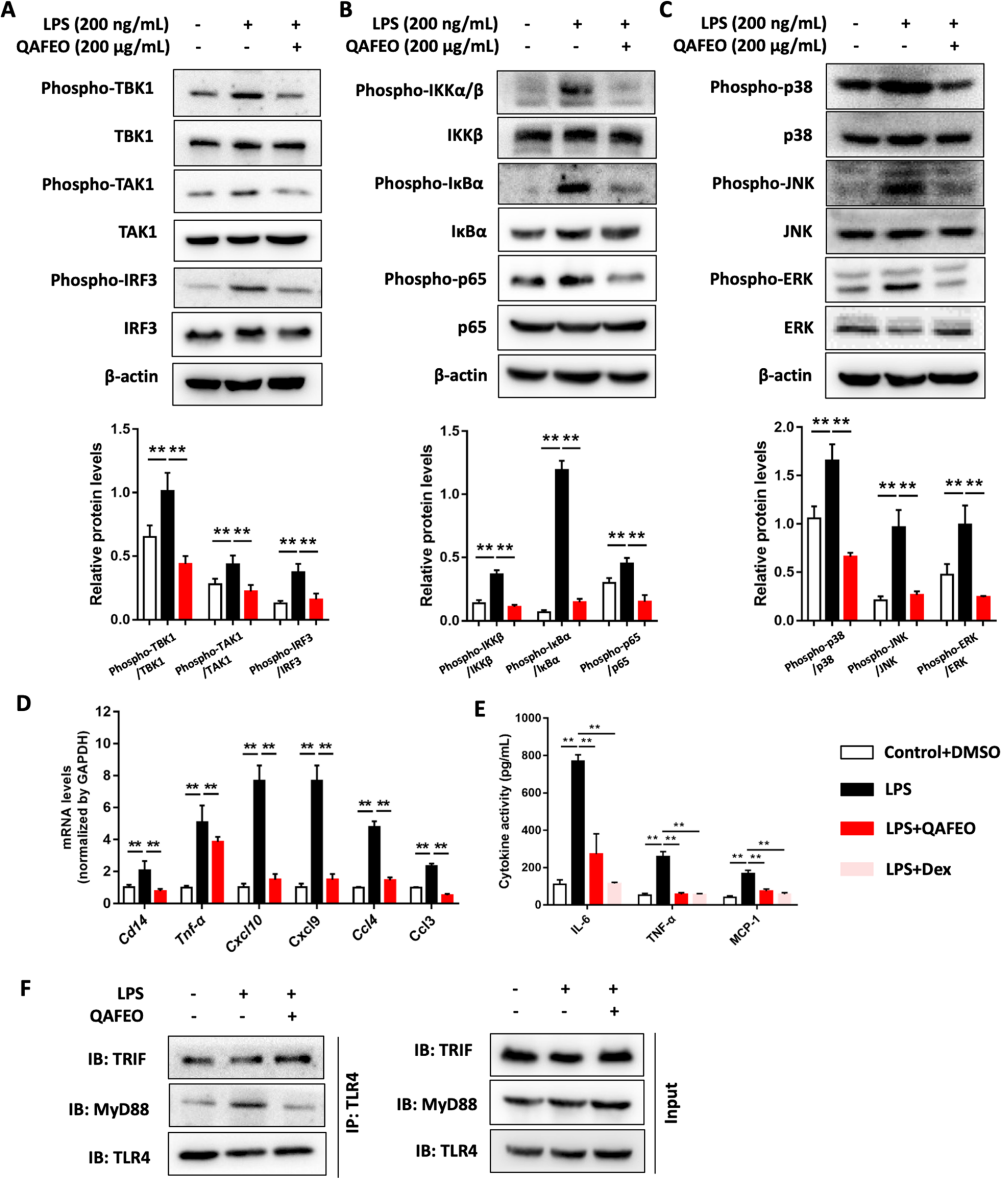
QAFEO regulates the LPS-mediated inflammatory signaling pathways in vitro. RAW 264.7 cells challenged by LPS (200 ng/mL) were treated with or without QAFEO (200 µg/mL, dissolved in DMSO) for 16 h. The protein abundance of **A** phospho-TBK1, TBK1, phospho-TAK1, TAK1, phospho-IRF3, IRF3, **B** phospho-IKKα/β, IKKβ, phospho-IκBα, IκBα, phospho-p65, p65 and **C** phospho-p38, p38, phospho-JNK, JNK, phospho-ERK, ERK were determined by western blotting. Quantification and statistical analysis of the protein expression. **D** RT-qPCR analysis of genes involved in the TLR4 pathways. Values were expressed as mean ± SD (n = 3). ^**^p < 0.01. **E** The concentration of IL-6, TNF-α and MCP-1 in the supernatant of the cell. Dex (Dexamethasone, 2 µM) was used as the positive drug. Data were expressed as the mean ± SD (n = 3). ^**^p < 0.01. **F** Co-immunoprecipitation (Co-IP) of TRIF/TLR4 and MyD88/TLR4 complexes in RAW 264.7 cells

To further validate the impact of QAFEO on the LPS-mediated inflammation, an in vitro model of inflammatory cells was established by stimulating RAW264.7 cells with LPS. In line with our findings from in vivo experiments, QAFEO was not cytotoxic (Additional file 1: Fig. S1C) and significantly curtailed the phosphorylation of TBK1, TAK1, and IRF3 in LPS-induced RAW 264.7 cells (Fig. 5A). Moreover, QAFEO notably controlled the activation of NF-κB and MAPK pathways, as evidenced by the inhibition of phosphorylation of IKK, IκBα, p65, p38, JNK, and ERK (Fig. 5B, C). Furthermore, various inflammatory factors such as *Cd14*, *Tnf-α*, *Ccl3*, *Ccl4*, and *Cxcl10* were obviously downregulated by QAFEO administration (Fig. 5D). Additionally, the administration of QAFEO resulted in a significant reduction in the levels of IL-6, TNF-α, and MCP-1 in the supernatant of RAW 264.7 cells induced by LPS. Notably, the observed suppressive effect of QAFEO on these pro-inflammatory cytokines closely resembled that of Dex (Dexamethasone, Fig. 5E). Next, we conducted co-immunoprecipitation (Co-IP) assays on TLR4 to probe the interaction between MyD88 and TRIF proteins in vitro. The results showed that LPS promotes TLR4 binding affinity to both MyD88 and TRIF, while QAFEO substantially reduced MyD88-TLR4 interaction levels without any impacts on TLR4-TRIF interactions (Fig. 5F).

### The compounds in QAFEO have binding affinity for LPS-mediated inflammatory pathway-related proteins

**Fig. 6:**
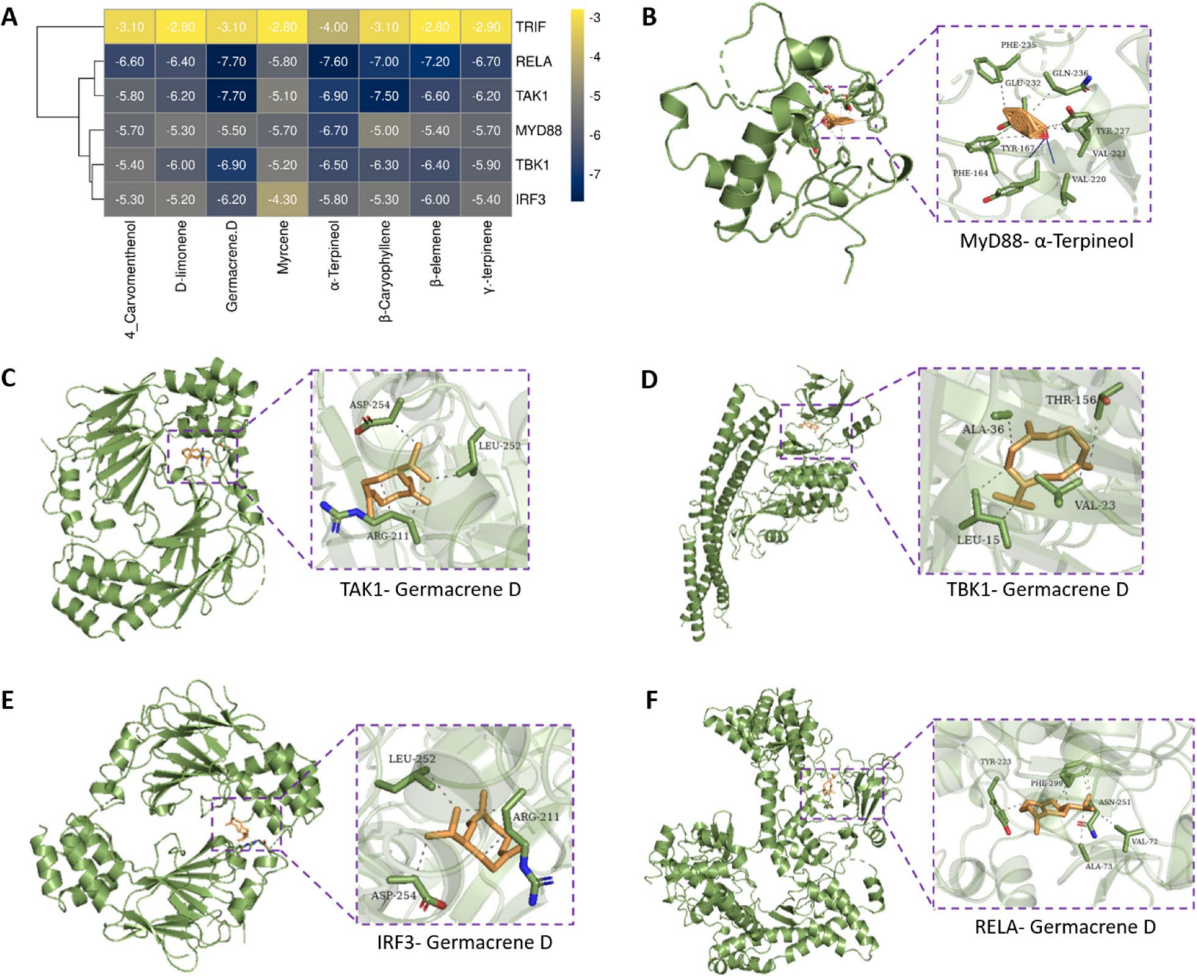
Molecular docking simulation of the binding between 8 key compounds of QAFEO and critical proteins involved in the LPS-mediated inflammatory pathway. **A** Binding energy score between 8 key compounds of QAFEO and critical proteins involved in the TLR4 pathway. **B**–**F** The representative binding poses of compounds in QAFEO and critical proteins involved in the TLR4 pathway

Using PubChem database, Openbable, pyMOL, and AutoDock software, we performed docking simulations of 8 compounds from QAFEO with TLR4 signaling pathway-related proteins including REAL (NF-κB p65), TAK1, MyD88, TBK1, IRF3 and TRIF. Docking scores lower than − 5 kcal/mol for the compound-target pairs are indicative of stable binding between the respective compounds and target proteins. The results demonstrated that all 8 compounds from QAFEO could bind to REAL, TAK1, MyD88, TBK1, and IRF3; however, none of these compounds could bind to TRIF (Fig. 6A). The binding poses of the 8 compounds in QAFEO with the targets displaying the most significant binding energy were shown in Fig. 6B–F.

## Discussion

Our study identifies, for the first time, a hepatoprotective effect of QAFEO against ALF in mice induced with LPS/D-GalN. Our findings elucidate the primary mechanism by which QAFEO exerts its prophylactic influence on ALF, whereby the LPS-mediated inflammatory signaling cascade is targeted to suppress the execution of inflammatory reactions.

Kupffer cells (KCs), alternatively referred to as hepatic macrophages, represent a distinct population of macrophages that reside within the liver. These cells are integral components of the mononuclear phagocyte system and differentiate from blood mononuclear cells by adhering to the hepatic sinusoid wall [12]. Activation of KCs can occur in response to bacterial LPS or cell superantigens, leading to the release of inflammatory mediators into the bloodstream. This activation subsequently triggers the activation of various inflammatory cells and contributes to the initiation and progression of liver inflammation [13]. KCs can be distinguished by the high expression levels of F4/80^+^ or CD68^+^ [14]. KCs have been shown to elicit a significant impact on ALF regulation. In ALF induced by acetaminophen, KCs exhibit dichotomous behavior in promoting liver injury repair and exacerbating APAP hepatotoxicity [15]. However, in LPS-induced ALF, KCs largely contribute to disease aggravation via LPS-mediated activation [16]. Our present investigation revealed a substantial increase in F4/80^+^ and CD68^+^ positive KCs in mice with LPS/D-GalN-induced ALF. Administration of QAFEO resulted in a marked decrease of KCs in the liver of ALF mice, concomitant with a pronounced reduction of proinflammatory cytokine levels in vivo and in vitro. The observations warrant the inference that QAFEO possesses the capacity to suppress hepatic inflammation in murine models of ALF.

LPS, a prominent constituent of the outer membrane in Gram-negative bacteria, is commonly released during bacterial growth and cell death [17]. In the context of systemic infections induced by Gram-negative bacteria, both LPS aggregates and intact bacterial cells are rapidly opsonized by LPS-binding protein (LBP), a serum protein synthesized in the liver. LBP plays a crucial role in facilitating the transfer of LPS to membrane-bound and soluble forms of CD14 [18]. Notably, the glycosylphosphatidylinositol-anchored mCD14 isoform is predominantly expressed in cells of the monocytic lineage and significantly enhances the sensitivity of myeloid cells to even minute quantities of LPS [17]. However, due to the absence of a transmembrane domain, mCD14 needs through toll-like receptors (TLRs) to initiate cellular signaling. TLRs are a group of transmembrane receptors within the pattern recognition receptor family that can recognize pathogen-associated molecular patterns (PAMPs) and damage-associated molecular patterns (DAMPs) [19]. TLRs exhibit high expression levels among immune and non-immune cells. In the present study, the results of liver transcriptomics showed that QAFEO significantly affected the response to LPS and TLRs signaling pathway, and down-regulated the expression of genes such as *Cd14*, *Tnf-α*, *Ccl4*. These results suggest that the effect of QAFEO in inhibiting liver inflammation may be related to the influence of LPS-mediated inflammatory signaling pathway.

TLR4, a member of the TLRs family [20], has been identified as a specific receptor for LPS leading to the induction and release of pro-inflammatory cytokines by monocytes and macrophages upon LPS stimulation, including but not limited to IL-1β, IL‐6, and TNF‐α, ultimately culminating in severe liver damage within a short timeframe [21–23]. Prior investigations have indicated that the induction of TLR4 leads to the modulation of pro-inflammatory cytokine expression, which is primarily achieved via MyD88-dependent or MyD88-independent signaling cascades [24]. TLR4 can interact with MyD88 to stimulate TAK1, resulting in the initiation of NF-κB and MAPK signaling cascades that regulate pro-inflammatory factor (*Tnf-α, Ccl4, Ccl3*) expression [25]. Moreover, TLR4 binding to TRIF induces activation of TBK1, thereby prompting IRF3 activation and the subsequent production of pro-inflammatory cytokines (*Cxcl9, Cxcl10, Il-11*) [26]. Our results suggested that QAFEO was found to exert a suppressive effect on the activation of TAK1, NF-κB, MAPK, TBK1, and IRF3 pathways within both in vivo and in vitro. These results collectively imply that QAFEO exhibits a systemic inhibition of the MyD88-dependent or MyD88-independent. Notably, QAFEO was found to hinder the interaction between TLR4 and MyD88, while showing no similar inhibitory effects towards the binding of TLR4 and TRIF. The molecular docking results showed that the entirety of the eight compounds present within QAFEO exhibited binding affinity towards MyD88, TAK1, TBK1 and IRF3. However, the docking analysis revealed a lack of binding interaction between these QAFEO compounds and TRIF. The present findings demonstrate that QAFEO exerts regulatory control over the MyD88-dependent signaling pathway via inhibition of TLR4 binding to MyD88, or by targeting proteins such as MyD88, TAK1, and NF-κB directly. However, the modulation of MyD88-independent signaling pathways is primarily achieved through direct effects on TBK1 and IRF3 pathways. The data presented in this study offer compelling evidence suggesting that QAFEO primarily exerts its effects on LPS-mediated inflammation by modulating both the MyD88-dependent and MyD88-independent signaling pathways. These findings contribute to our understanding of the molecular mechanisms underlying the anti-inflammatory properties of QAFEO. Nonetheless, further investigations are warranted to unravel the mechanistic basis of these findings.

Taken together, the results of our study support the notion that QAFEO exerts a hepatoprotective effect through its modulation of the LPS-mediated inflammatory signaling pathway. Our findings may pave the way for future clinical applications of QAFEO as well as the development of novel liver-protective agents.

### Supplementary Information

**Additional file 1. Fig. S1.** QAFEO toxicity test in vivo and in vitro. **A**, **B** The male BALB/c mice were randomly divided into three groups: control group (oral saline administration for five days), QAFEO-50 group (oral administration of 50 mg/kg/day QAFEO for five days), and QAFEO-100 group (oral administration of 100 mg/kg/day QAFEO for five days). **A **H&E staining of liver sections. **B** The levels of ALT and AST from the serum and the livers. Data were expressed as the mean ± SD (n=8). **C** RAW 264.7 cells were treated with different doses of QAFEO (0, 50, 100, 200 μg/mL) for 16 h. LDH kit was used to detect cell death. Data were expressed as the mean ± SD (n=5).

## Data Availability

The data supporting the findings of this study are available upon reasonable request from the corresponding author.

## Abbreviations

ALF: Acute liver failure
ALT: Alanine aminotransferase
ANOVA: Analysis of variance
AST: Aspartate aminotransferase
Co-IP: Co-immunoprecipitation
DAMPs: Damage-associated molecular patterns
D-GalN: D-galactosamine
DEGs: Differentially expressed genes
Dex: Dexamethasone
DMSO: Dimethyl sulfoxide
EDTA: Ethylene diamine tetraacetic acid
ERK: Extracellular regulated protein kinases
FBS: Fetal bovine serum
PMSF: Phenylmethylsulfonyl fluoride
GC–MS/MS: Gas chromatography-tandem mass spectrometry
GO: Gene ontology
H&E: Hematoxylin and eosin
IκB: Inhibitor of NF-κB
IKK: Inhibitor of kappa B kinase
i.p.: Intraperitoneal injection
IRF3: Interferon regulatory Factor 3
KCs: Kupffer cells
JNK: C-Jun N-terminal kinase
KEGG: Kyoto Encyclopedia of Genes and Genomes
LPS: Lipopolysaccharide
LBP: LPS-binding protein
LPS/D-GalN: Lipopolysaccharide and D-galactosamine
MAPK: Mitogen-activated protein kinase
NF-κB: Nuclear factor kappa-B
PAMPs: Pathogen-associated molecular patterns
PVDF: Polyvinylidene fluoride
QAF: Quzhou Aurantii Fructus
QAFEO: Quzhou Aurantii Fructus essential oil
RNA-seq: RNA-sequencing
RT-qPCR: Real-time quantitative polymerase chain reaction
SDS-PAGE: Sodium dodecyl sulfate polyacrylamide gel electrophoresis
SD: Standard deviation
TAK1: TGF beta-activated kinase 1
TBK1: Recombinant TANK binding kinase 1
TLR4: Toll-like receptor 4
TLRs: Toll-like receptors
TRIF: TIR domain-containing adaptor inducing interferon-β

## References

[1] Stravitz RT, Lee WM (2019). Acute liver failure. Lancet.

[2] Liu Y, Lou G, Li A, Zhang T, Qi J, Ye D, Zheng M, Chen Z (2018). AMSC-derived exosomes alleviate lipopolysaccharide/d-galactosamine-induced acute liver failure by mir-17-mediated reduction of TXNIP/NLRP3 inflammasome activation in macrophages. EBioMedicine.

[3] Dorman HJ, Deans SG (2000). Antimicrobial agents from plants: antibacterial activity of plant volatile oils. J Appl Microbiol.

[4] Silva GL, Luft C, Lunardelli A, Amaral RH, Melo DA, Donadio MV, Nunes FB, de Azambuja MS, Santana JC, Moraes CM, Mello RO, Cassel E, Pereira MA (2015). De Oliveira, antioxidant, analgesic and anti-inflammatory effects of lavender essential oil. An Acad Bras Cienc.

[5] Sun Z, Wang H, Wang J, Zhou L, Yang P (2014). Chemical composition and anti-inflammatory, cytotoxic and antioxidant activities of essential oil from Leaves of Mentha piperita grown in China. PLoS ONE.

[6] Gao L, Zhang H, Yuan CH, Zeng LH, Xiang Z, Song JF, Wang HG, Jiang JP (2022). Citrus aurantium ‘Changshan-huyou’-An ethnopharmacological and phytochemical review. Front Pharmacol.

[7] Bai YF, Wang SW, Wang XX, Weng YY, Fan XY, Sheng H, Zhu XT, Lou LJ, Zhang F (2019). The flavonoid-rich Quzhou Fructus Aurantii extract modulates gut microbiota and prevents obesity in high-fat diet-fed mice. Nutr Diabetes.

[8] Wang SW, Lan T, Zheng F, Lei MK, Zhang F (2021). [Effect of extract of Quzhou Aurantii Fructus on hepatic inflammation and NF-kappaB/NLRP3 inflammasome pathway in CCl_4-induced liver fibrosis mice]. Zhongguo Zhong Yao Za Zhi.

[9] Wang SW, Lan T, Sheng H, Zheng F, Lei MK, Wang LX, Chen HF, Xu CY, Zhang F (2021). Nobiletin alleviates non-alcoholic Steatohepatitis in MCD-Induced mice by regulating macrophage polarization. Front Physiol.

[10] Wang SW, Lan T, Chen HF, Sheng H, Xu CY, Xu LF, Zheng F, Zhang F (2022). Limonin, an AMPK activator, inhibits hepatic lipid Accumulation in High Fat Diet Fed mice. Front Pharmacol.

[11] Wang SW, Wang W, Sheng H, Bai YF, Weng YY, Fan XY, Zheng F, Zhu XT, Xu ZC, Zhang F (2020). Hesperetin, a SIRT1 activator, inhibits hepatic inflammation via AMPK/CREB pathway. Int Immunopharmacol.

[12] Zigmond E, Samia-Grinberg S, Pasmanik-Chor M, Brazowski E, Shibolet O, Halpern Z, Varol C (2014). Infiltrating monocyte-derived macrophages and resident kupffer cells display different ontogeny and functions in acute liver injury. J Immunol.

[13] Tacke F (2017). Targeting hepatic macrophages to treat liver diseases. J Hepatol.

[14] Weston CJ, Zimmermann HW, Adams DH (2019). The role of myeloid-derived cells in the progression of Liver Disease. Front Immunol.

[15] Yang T, Wang H, Wang X, Li J, Jiang L (2022). The dual role of innate immune response in acetaminophen-induced liver injury. Biology (Basel).

[16] Tsutsui H, Nishiguchi S (2014). Importance of Kupffer cells in the development of acute liver injuries in mice. Int J Mol Sci.

[17] Di Lorenzo F, Duda KA, Lanzetta R, Silipo A, De Castro C, Molinaro A (2022). A journey from structure to function of bacterial lipopolysaccharides. Chem Rev.

[18] Schroder NW, Morath S, Alexander C, Hamann L, Hartung T, Zahringer U, Gobel UB, Weber JR, Schumann RR (2003). Lipoteichoic acid (LTA) of Streptococcus pneumoniae and Staphylococcus aureus activates immune cells via toll-like receptor (TLR)-2, lipopolysaccharide-binding protein (LBP), and CD14, whereas TLR-4 and MD-2 are not involved. J Biol Chem.

[19] Squillace S, Salvemini D (2022). Toll-like receptor-mediated neuroinflammation: relevance for cognitive dysfunctions. Trends Pharmacol Sci.

[20] Johnson GB, Brunn GJ, Platt JL (2004). Cutting edge: an endogenous pathway to systemic inflammatory response syndrome (SIRS)-like reactions through toll-like receptor 4. J Immunol.

[21] Maes M, Vinken M, Jaeschke H (2016). Experimental models of hepatotoxicity related to acute liver failure. Toxicol Appl Pharmacol.

[22] Lv H, Yang H, Wang Z, Feng H, Deng X, Cheng G, Ci X (2019). Nrf2 signaling and autophagy are complementary in protecting lipopolysaccharide/d-galactosamine-induced acute liver injury by licochalcone A. Cell Death Dis.

[23] Liu Y, Liu N, Liu Y, He H, Luo Z, Liu W, Song N, Ju M (2022). Ginsenoside Rb1 reduces D-GalN/LPS-induced Acute Liver Injury by regulating TLR4/NF-kappaB signaling and NLRP3 inflammasome. J Clin Transl Hepatol.

[24] Luo W, Lin K, Hua J, Han J, Zhang Q, Chen L, Khan ZA, Wu G, Wang Y, Liang G (2022). Schisandrin B attenuates Diabetic Cardiomyopathy by Targeting MyD88 and inhibiting MyD88-Dependent inflammation. Adv Sci (Weinh).

[25] Zhu W, Luo W, Han J, Zhang Q, Ji L, Samorodov AV, Pavlov VN, Zhuang Z, Yang D, Yin L, Huang L, Liang G, Huh JY, Wang Y (2023). Schisandrin B protects against LPS-induced inflammatory lung injury by targeting MyD88. Phytomedicine.

[26] Ko R, Seo J, Park H, Lee N, Lee SY (2022). Pim1 promotes IFN-beta production by interacting with IRF3. Exp Mol Med.

