# Supplementary material for: Essential oil extracted from Quzhou Aurantii Fructus prevents acute liver failure through inhibiting lipopolysaccharide-mediated inflammatory response
Source: Nat Prod Bioprospect. 2023 Oct 7;13(1):36. doi: 10.1007/s13659-023-00398-9 (PMC10560171; doi:10.1007/s13659-023-00398-9)
Supplement: Supplementary file 1 — Additional file 1. Fig. S1. QAFEO toxicity test in vivo and in vitro. A, B The male BALB/c mice were randomly divided into three groups: control group (oral saline administration for five days), QAFEO-50 group (oral administration of 50 mg/kg/day QAFEO for five days), and QAFEO-100 group (oral administration of 100 mg/kg/day QAFEO for five days). A H&E staining of liver sections. B The levels of ALT and AST from the serum and the livers. Data were expressed as the mean ± SD (n=8). C RAW 264.7 cells were treated with different doses of QAFEO (0, 50, 100, 200 μg/mL) for 16 h. LDH kit was used to detect cell death. Data were expressed as the mean ± SD (n=5). [file 13659_2023_398_MOESM1_ESM.docx]

Additional file 1: Figures


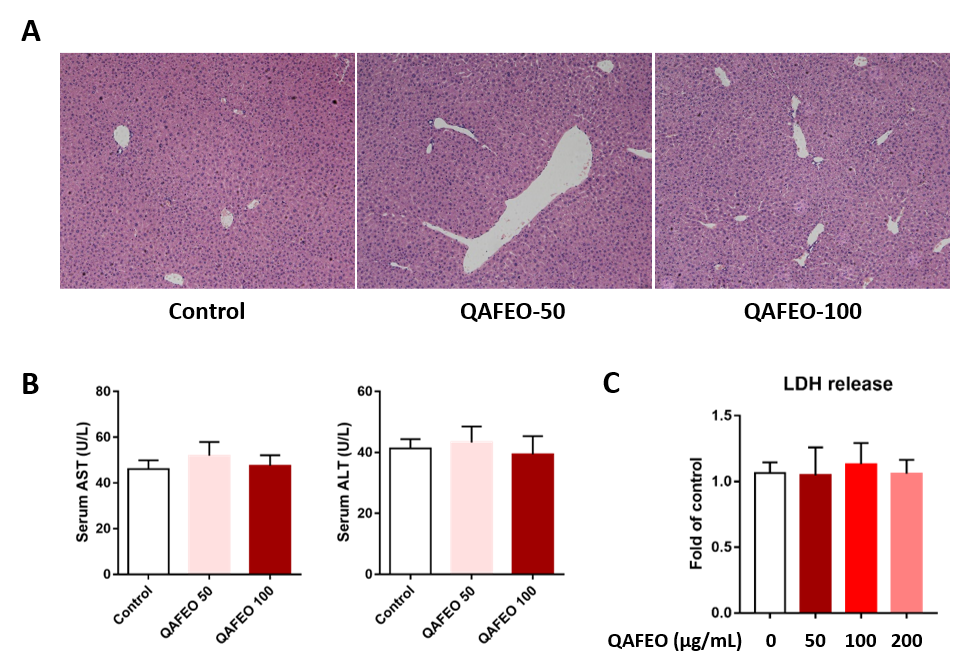


**Fig. S1 QAFEO toxicity test *in vivo* and *in vitro*.** **(A and B)** The male BALB/c mice were randomly divided into three groups: control group (oral saline administration for five days), QAFEO-50 group (oral administration of 50 mg/kg/day QAFEO for five days), and QAFEO-100 group (oral administration of 100 mg/kg/day QAFEO for five days). **(A)** H&E staining of liver sections. **(B)** The levels of ALT and AST from the serum and the livers. Data were expressed as the mean ± SD (n=8). **(C)** RAW 264.7 cells were treated with different doses of QAFEO (0, 50, 100, 200 μg/mL) for 16 h. LDH kit was used to detect cell death. Data were expressed as the mean ± SD (n=5).
